# Non-Invasive Diagnostic System Based on Light for Detecting Early-Stage Oral Cancer and High-Risk Precancerous Lesions—Potential for Dentistry

**DOI:** 10.3390/cancers12113185

**Published:** 2020-10-29

**Authors:** Seiko Tatehara, Kazuhito Satomura

**Affiliations:** Department of Oral Medicine and Stomatology, School of Dental Medicine, Tsurumi University, 2-1-3 Tsurumi, Tsurumi-ku, Yokohama City, Kanagawa 230-8501, Japan; tatehara-s@tsurumi-u.ac.jp

**Keywords:** chemiluminescence, autofluorescence, photodynamic diagnosis (PDD), oral cancer, oral epithelial dysplasia (OED), oral potentially malignancy disorders (OPMDs), protoporphyrin IX, fluorescence

## Abstract

**Simple Summary:**

The early detection of oral cancer and oral potentially malignant disorders can facilitate minimum intervention and subsequent improvements in prognosis and quality of life after treatment. Recently, several non-invasive adjunctive fluorescence-based detection systems have improved the accuracy of detection and diagnosis of oral cancer and oral potentially malignant disorders. This article summarizes current knowledge about fluorescence-based diagnostic methods and discusses their benefits and drawbacks from a clinical viewpoint.

**Abstract:**

Oral health promotion and examinations have contributed to the early detection of oral cancer and oral potentially malignant disorders, leading to the adaptation of minimally invasive therapies and subsequent improvements in the prognosis/maintenance of the quality of life after treatments. However, the accurate detection of early-stage oral cancer and oral epithelial dysplasia is particularly difficult for conventional oral examinations because these lesions sometimes resemble benign lesions or healthy oral mucosa tissues. Although oral biopsy has been considered the gold standard for accurate diagnosis, it is deemed invasive for patients. For this reason, most clinicians are looking forward to the development of non-invasive diagnostic technologies to detect and distinguish between cancerous and benign lesions. To date, several non-invasive adjunctive fluorescence-based detection systems have improved the accuracy of the detection and diagnosis of oral mucosal lesions. Autofluorescence-based systems can detect lesions as a loss of autofluorescence through irradiation with blue-violet lights. Photodynamic diagnosis using 5-aminolevulinic acid (ALA-PDD) shows the presence of very early oral cancers and oral epithelial dysplasia as a red fluorescent area. In this article, currently used fluorescence-based diagnostic methods are introduced and discussed from a clinical point of view.

## 1. Introduction

Oral cancer originating from the lip and oral cavity has been ranked as the 16th most common cancer worldwide, but the global incidence rate has seen a decline. In South and Southeast Asia in particular, alcohol intake and the use of tobacco including betel quid, which are risk factors for oral cancer, are high. Thus, the incidence rate remains elevated. In addition, human papillomavirus (HPV) is a high risk factor, and the incidence of HPV-related oral cancer has increased in specific areas [1]. Meanwhile, the incidence of oral cancer has increased in younger generations and women [1]. The 5-year overall survival rate is about 50%, which is poorer than other cancers because most cases are diagnosed at an advanced stage [2]. When oral cancer is detected at an early stage (T1 or early T2), the survival rate can be increased by as much as 90% by radical treatments, surgery, or radiotherapy, and reduced oral dysfunction is observed after treatment [3]. In cases diagnosed with locally advanced disease, there is a limit to preserving oral function and maintaining the quality of life of patients because dysphagia, dysarthria, psychological distress, and cosmetic disturbance are often caused by a large resection and reconstruction. Therefore, clinicians should strategically focus on the detection of early-stage oral cancer to improve the survival rate [4].

In 2017, the World Health Organization (WHO) stated that oral precancerous lesions and oral precancerous conditions are called oral potentially malignant disorders (OPMDs) [5]. OPMDs often include histopathologically oral epithelial dysplasia (OED) [6], which has been defined as the presence of specific epithelial architectural and cytological changes and graded as mild, moderate, and severe according to the depth and severity of changes [5]. Mehanna et al. reported in a 2009 meta-analysis that the transformation rate is 12.1% for all OEDs, 10.3% for mild to moderate dysplasia, and 24.1% for severe dysplasia and carcinoma in situ (CIS) [7]. Moreover, Sperandio et al. reported that the transformation rate was around 0.012% for non-dysplastic lesions, 6% for mild, 18% for moderate, and 39% for severe dysplasia [8]. Warnakulasuriya et al. indicated that the transformation rates for mild, moderate, and severe dysplasia were 4.8%, 15.7%, and 26.7 %, respectively, and annual transformation rates for moderate and severe dysplasia were 1.8% and 5.6%, respectively [9]. From these results (Table 1), OPMDs including histopathologically moderate and severe OED are considered as high-risk OPMDs, which have a high potential to transform into cancer [7,8,9,10]. Therefore, clinicians should also detect early high-risk OPMDs for the minimum intervention or monitoring of lesions [10,11].

The conventional oral examination (COE) has been identified as a standard diagnostic procedure for clinicians in detecting oral mucosal changes by inspection and palpation [12,13]. Epstein et al. conducted a systematic review to assess the accuracy of the COE in detecting OED and oral squamous cell carcinoma (OSCC) and suggested that the sensitivity and specificity of COEs were 93% and 31%, respectively [12]. This suggested that the number of false-positive cases was large, indicating that clinicians likely diagnosed several suspicious lesions as malignant lesions.

Considering this point, it can be concluded that the COE alone cannot accurately detect and discriminate between OEDs or OSCC and other lesions or classify high-risk and low-risk OPMDs, even though oral cancer specialists perform the COE [7,14,15]. OED, CIS, and early OSCC often exist as superficial, minute, or minor lesions as normal mucosa, whereas frictional keratosis, leukoplakia, and leukoedema clinically resemble high-risk OPMDs [16,17]. Therefore, the development of an easy, non-invasive, and accurate diagnostic tool in detecting early-stage OSCC and high-risk OPMDs is urgently required [13]. Recently, some fluorescence-based methods and optical diagnostic devices have been developed and applied on an outpatient basis in clinic settlings [18,19,20,21,22,23,24,25,26,27,28,29,30,31,32,33,34,35,36,37,38,39,40,41,42,43,44,45,46,47].

In this article, some light-based diagnostic technologies for early-stage OSCC and high-risk OPMDs are introduced, with a special emphasis on photodynamic diagnosis using 5-aminolevulinic acid (ALA-PDD), and discussed from a clinical viewpoint.

## 2. Examination under Chemiluminescent Illumination

**Figure 1:**
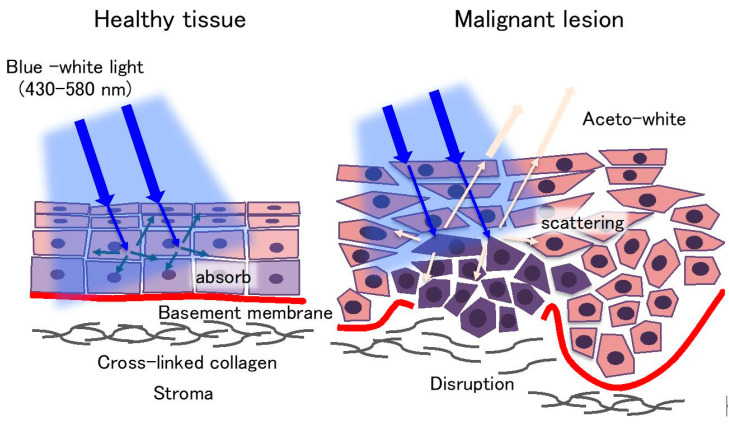
The mechanism underlying the chemiluminescent light system. Healthy tissues appear as a dark blue hue, whereas abnormal lesions display an “aceto-white.” This appearance is caused by the differences in backscattering light between normal mucosa and abnormal lesions.

The ViziLite^®^ (Zila Pharmaceuticals, Inc., Phoenix, AZ, USA) system consists of a disposable chemiluminescent light source and a 1% acetic acid solution [18,19]. The disposable chemiluminescent light source is formed by an outer shell of flexible plastic containing acetylsalicylic acid and an inner vial of fragile glass containing hydrogen peroxide. Before the examination, the disposable chemiluminescent light source is bent to break the inner vial, thereby starting the chemical reaction between acetylsalicylic acid and hydrogen peroxide, which produces a bluish-white light (wavelength ranging from 430 to 580 nm) for about 10 min [18,19]. In clinical use, an oral rinse with a 1% acetic acid solution is performed for 30–60 s to remove the debris and glycoprotein layer on lesions, and then an oral examination is performed under blue-white light. Normal mucosa appears as a dark blue hue, whereas abnormal lesions with abnormal nuclei, high nuclear/cytoplasmic ratios, excess keratinization, and predominant inflammatory infiltration display an “aceto-white” (Figure 1) [18,19]. This different appearance is caused by the differences in backscattering light between normal mucosa and abnormal lesions (Figure 1). From all reports on chemiluminescent light systems published from 2007 to 2018, the sensitivity and specificity for the detection of OSCC and OPMDs were determined ranging between 0–100% and 0–27.8%, respectively [18,19,20,21,22,23,24,25,26]. Most reports demonstrated that the sensitivity for the detection of OSCC and OED was more than 70%. Other studies reported that chemiluminescent examinations could improve brightness and sharpness of margins in identified lesions [18,19]. In particular, in white and keratotic lesions such as leukoplakia, this technology could enhance their visualization and sharpness [20]. Interestingly, Vashisht et al. reported that ViziLite could detect early epithelial dysplastic changes that clinically appeared as normal oral mucosa [21]. However, red malignant lesions such as erythroplakia [20], inflammatory [22], and erosive lesions [23] were diagnosed as false-negative cases. Of the 12 reports, 9 reports indicated that the specificity was less than 50% [18,19,20,22,24,25,26], suggesting high false-positive rates. One explanation is that this system tends to judge all keratotic lesions as malignancy lesions. To improve the low specificity, Kämmerer et al. applied a chemiluminescent light system combined with a toluidine blue staining and indicated that the sensitivity reduced by 20% (100% to 80%) and specificity increased by 67.5% (30% to 97.5%) compared with in the chemiluminescent light system alone [22]. Finally, these previous studies demonstrated that the chemiluminescent light system alone could not provide any better outcomes than COEs [24], nor discriminate between benign or inflammatory and premalignant or malignant lesions.

## 3. Autofluorescence Imaging

**Figure 2:**
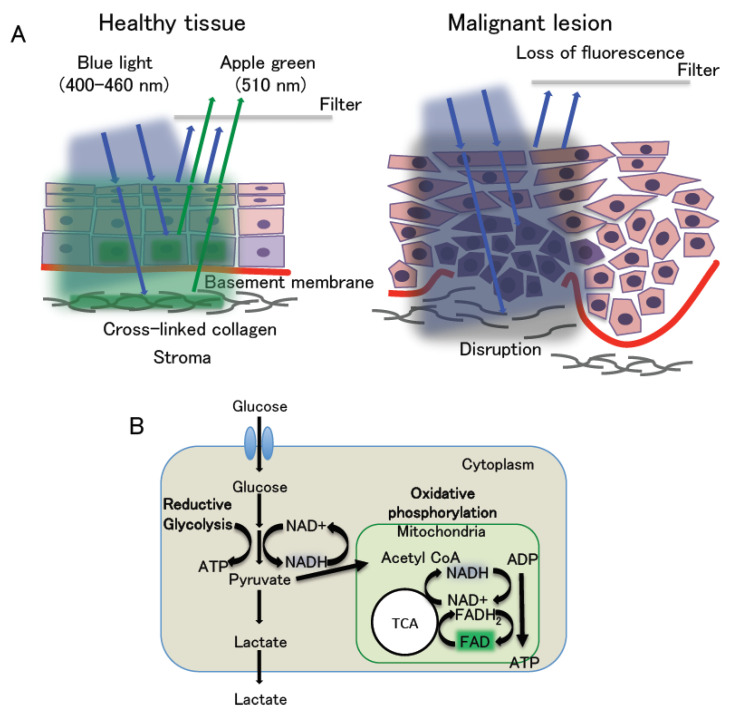
The mechanism underlying autofluorescence-based diagnosis. (**A**) Under blue light at 400–460 nm, healthy tissues exhibit apple green fluorescence (510 nm), while malignant lesions display a loss of autofluorescence. This is related to the redox ratio, autofluorescence of cross-link collagen, and the filtering effect. (**B**) Cell metabolism. This phenomenon is related to the Warburg effect. Malignant lesions dominate reductive glycolysis, leading to the decrease in the redox ratio. TCA: tricarboxylic acid cycle.

Autofluorescence imaging has been attracting increasing attention; in fact, it has been applied in clinical practice worldwide using several devices, such as the VELscope (LED Dental, Atlanta, GA, USA), Identafi (StarDental-DentalEZ, Englewood, CO, USA), and IllumScan (Shofu, Kyoto, Japan). These systems provide irradiation with blue light with a wavelength of 400–460 nm for excitation, and tissue autofluorescence is visualized with a maximum wavelength of 510 nm through a 475-nm long-pass filter mounted in the devices [27]. The long-pass filter transmits fluorescence with wavelengths exceeding 480 nm and blocks blue excitation fluorescence with wavelengths of less than 470 nm. These systems display healthy mucosa tissues as apple green fluorescence, which is termed autofluorescence. In contrast, OSCC and OED are visualized as a loss of autofluorescence indicated by black areas (Figure 2) [18,19,27]. Autofluorescence relates to certain endogenous fluorophores including respiratory chain (mitochondrial) enzymes in healthy cells such as nicotinamide adenine dinucleotide (NADH) and flavin adenine dinucleotide (FAD), and normal collagen (and elastin) cross-linked structures. The two enzymes, NADH and FAD, form redox pairs. NADH is fluorescent in its reduced form but not when it is oxidized to NAD+. NADH exhibits excitation fluorescence at 350 nm and emission fluorescence at 460 nm of maximum wavelength. Meanwhile, FAD is fluorescent in its oxidized form and loses its fluorescence when reduced (FADH_2_). FAD has excitation and emission fluorescence maxima at 450 and 535 nm, respectively [28]. The fluorescence intensity ratio of FAD (oxidized form) to NADH (reduced form), called the “redox ratio”, changes according to the redox state of the tissue, demonstrating whether the cell metabolism is more oxidative phosphorylation or reductive glycolysis [28]. The redox ratio is closely related to the Warburg effect, which means that oxidative phosphorylation dominates in normal cells, whereas reductive glycolysis is most active in malignant cells [29] (Figure 2). In short, healthy tissues have a high redox ratio, whereas malignant lesions have a low redox ratio. Furthermore, normal collagen emits fluorescence maxima at 535 nm under blue light at 400–460 nm, but not when a cross-linked collagen is disrupted. Thus, healthy tissues appear apple green and malignant lesions display a loss of autofluorescence, caused by the redox ratio, fluorescence of collagen, and the filtering effect (Figure 2). Previous reports published from 2007 to 2018 indicated that the sensitivity and specificity for OSCC and OED were 30–100% and 6–100%, respectively [18,19,30,31,32,33,34,35]. These results indicated that autofluorescence-based devices could detect OSCC and OED, but the number of false positives was found to be larger than expected [18,19,30,31,32], suggesting that this assay has difficulty in distinguishing between malignant and benign lesions. For this reason, autofluorescence imaging identifies infection sites and erythematous lesions, such as gingivitis, pigmentation, ulceration, or irritation, as fluorescent visualization loss (FVL) [18,30,33]. In addition, the excitation fluorescence (400–460 nm) only reaches a depth of 400–600 nm from the surface of the lesion. Thus, this system cannot detect malignant lesions covered by a thick keratin layer or collagen fibers [14,30]. Moreover, the autofluorescence system visualizes the hemorrhagic lesion as FVL; this is because the hemoglobin in the blood highly absorbs the blue light. This system can also be subjected to the superficial condition of mucosal lesions [33]. Regarding OED, most OEDs are visualized as FVL; thus, they cannot be classified into high or low grades [30]. Hanken et al. suggested that the high false-positive rates might lead to overdiagnosis and overtreatment [31]. In the case of hemorrhagic or thick lesions, narrow band imaging (NBI) is an optical diagnostic technique that might overcome these problems, as it can detect alternative capillary structures in the surface lesions via irradiation with two excitation fluorescence wavelengths of 390–445 and 530–550 nm [45].

This system is also considered a subjective examination as it depends on the experience of individual examiners in judging whether the mucosal lesions are visualized as FVL. Rana et al. and Hanken et al. compared the COE alone and the combination of the autofluorescence device and COE and concluded that the combination improved the sensitivity but lowered the specificity for OSCC and oral premalignant lesions, including OED [31,34]. To address this problem, Yamamoto et al. and Huang et al. performed a quantitative analysis for the difference of the luminance ratio or intensity between malignant and healthy lesions, which improved the sensitivity and specificity [32,35]. Therefore, the autofluorescence-based system is capable of assisting the discrimination of OSCC and OED from benign lesions when combined with the COE and quantitative analysis of autofluorescence. However, this system cannot discriminate between high-risk and low-risk dysplasia.

## 4. Photodynamic Diagnosis

Photodynamic diagnosis (PDD) is a technology that enables the identification of pathological lesions as fluorescent areas excited by the irradiation of light with a specific wavelength after the local or systemic administration of a photosensitizer (PS) or pro-PS drug. In the field of head and neck cancer, including those of the oral cavity, Leonard et al. applied PDD to detect neoplasia of the oral cavity, pharynx, hypopharynx, and larynx in 1971 [48]. In their study, lesions were visualized as red fluorescence by ultraviolet light (405 nm) after the intravenous application of a PS. Following intravenous injection, PSs are incorporated into normal and cancer cells and transported to mitochondria. In normal cells, PSs are promptly metabolized through heme biosynthesis, whereas they accumulate in cancer cells [49]. As the time required until the difference in the PS concentration between normal and malignant tissue reaches a peak differs according to administration routes and the kinds of PS, the timing for examination should be carefully and adequately determined.

Porfimer sodium (Photofrin^®^, Axcan Pharma, Inc, Mont-Saint-Hilaire, QC, Canada) and temoporfin (Foscan^®^, Biolitec Pharma Ltd., Jena, Germany) are applied for head and neck cancers [49]. Photofrin, a hematoporphyrin derivative, is known to selectively accumulate within 48 h after intravenous administration [50]. However, Photofrin was maintained in the body for at least 2 weeks or several months, leading to skin phototoxic reactions when exposed to the sun [49]. Therefore, after PS administration, patients must avoid solar irradiation until the drug is excreted from the body. Foscan, as a second-generation PS, was developed to overcome some drawbacks of Photofrin, such as accumulation in normal cells and slow excretion from the body [49]. Foscan accumulates more rapidly in cancer cells and has a lower incidence of skin photosensitivity compared with Photofrin because of its rapid elimination from in the body. Currently, Photofrin and Foscan are used for photodynamic therapy rather than PDD [49].

### 4.1. 5 Aminolevulinic Acid and the Mechanism of ALA-PDD

As an alternative to hematoporphyrin derivatives, 5-Aminolevulinic acid (ALA) has been used as an ideal pro-PS for PDD. Kennedy et al. and Peng et al. confirmed that ALA was safe and effective for PDD [51,52].

**Figure 3:**
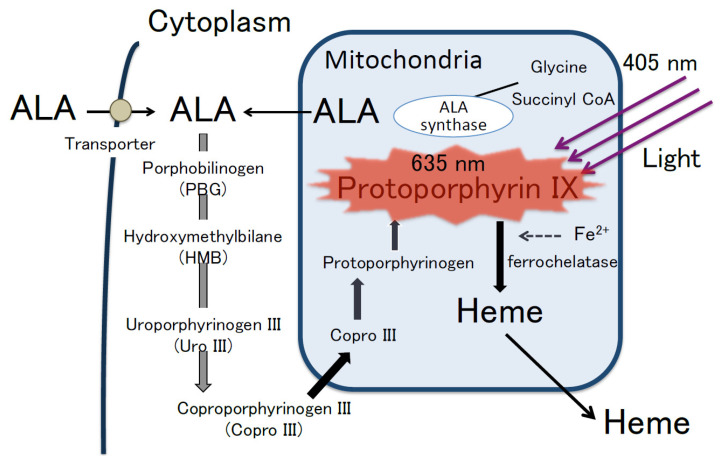
Heme biosynthesis pathway and mechanism of ALA-PDD. PpIX accumulates at higher levels in malignant cells than in normal cells. PpIX emit red fluorescence (635 nm) by blue light (405 nm). Modified from [53].

ALA is a water-soluble and natural amino acid that is synthesized from glycine and succinyl coenzyme A in the mitochondria; this is used for the synthesis of hemoglobin in erythrocytes through the biosynthetic pathway for heme (Figure 3) [53]. The fluorescent molecule protoporphyrin IX (PpIX) is converted from ALA by some mitochondrial enzymes during heme biosynthesis [54]. Free porphyrin metabolites, such as PpIX and heme tend to damage cells by inducing oxidative stress [54]. To prevent cell damage, the level of free porphyrin metabolites is regulated through a negative feedback mechanism that inhibits heme biosynthesis and stimulates heme degradation [54]. In addition, excess heme and PpIX are excreted from cells through membrane transporters, such as the ATP-binding cassette sub-family G (ABCG) 2 and cell membrane-associated molecules involved in exocytosis such as dynamin 2 [53,54,55].

After administration, exogenous ALA is transported to the cytoplasm by membrane transporters such as PEPT 1/2 and converted to PpIX through the heme biosynthetic pathway [53,54], and the level of PpIX in normal cells is regulated via a negative feedback loop in order to ensure that it does not accumulate in these cells. In contrast, PpIX accumulates at high levels in cancer cells [53,54]. Although the detailed mechanism of PpIX accumulation in cancer cells remains unclear, possible causes include reduced ferrochelatase activity through the heme biosynthetic pathway, impaired intracellular negative feedback mechanisms, and/or impairment of its excretion [53,54,56].

PpIX has the Soret-band around 405 nm. Thus, PpIX absorbs blue light (405 nm) and excites red fluorescence (635 nm) [57]. Briefly, cancer cells with accumulated PpIX emit red fluorescence (635 nm) following irradiation with blue light (375–440 nm) (Figure 3) [53,56]. Therefore, photodynamic diagnosis using 5-aminolevulinic acid (ALA-PDD) enables the differential visualization of cancer cells. However, the time when malignant lesions are selectively visualized as red fluorescence is limited. After ALA administration, the concentration of PpIX in malignant lesions gradually reaches a peak and declines to the same level in healthy tissues in several hours, whereas the concentration of PpIX in healthy tissues remains at the far lower level than in malignant lesions [56]. Consequently, the optimal time for examination is when the PpIX concentration in malignant lesion reaches a peak. Furthermore, blue light also excites endogenous fluorophores in normal tissues, resulting in the emission of green autofluorescence (510 nm). In particular, ALA-PDD clearly identifies malignant lesions as red fluorescence, whereas normal tissues as green autofluorescence.

### 4.2. ALA Administration Route

The routes of administration of ALA are primarily oral and topical administration for ALA-PDD [56]. The oral administration has been used in the fields of brain, bladder, and digestive organ cancer [56]. In the oral cavity, most previous reports used a topical administration (Table 1). Leunig et al. found that the peak ratio of the intensity of PpIX fluorescence between malignant lesions and healthy tissues was 12.5 at 1.5 h after the topical administration of 0.4% ALA solution [38,39]. Therefore, the optimal time for ALA-PDD is thought to be at 1–2 h after topical administration. In addition, they analyzed the distribution and penetration depth of PpIX in oral cancer tissues after the topical application [39]. The PpIX fluorescence was found to accumulate in the dysplastic epithelium but not in the stroma. Moreover, the penetration depth of ALA is limited to no more than 1 mm from the superficial epithelium. These reports may demonstrate the possibility that the topical application could be used for the detection of oral cancer at an early stage such as CIS and OED only if the duration after administration and the dose of ALA are selected carefully and adequately.

### 4.3. Concentration of ALA

Most clinicians in the head and neck cancer field have applied the topical administration of 0.4–1.0% ALA solutions and incubated for 1–2.5 h to accumulate PpIX in malignant cells. They suggested that ALA-PDD with topical application of 0.4% ALA could facilitate the visualization of OSCC and OEDs. Therefore, the use of low ALA concentrations must be sufficient for visualization of the superficial mucosa lesion. Furthermore, the usage of as low concentration of ALA as possible would be beneficial to avoid potential side effects, such as skin phototoxicity.

### 4.4. Side Effects of ALA

The side effects of ALA depend on the route of administration. Oral administration is reported to cause mild gastrointestinal disorders such as nausea and vomiting [56,58]. Although free PpIX and heme can be eliminated from the body within 6 h after application, exposure to daylight causes transient erythema (6%) [53,56,59]. Meanwhile, the topical application does not cause any serious side effects. The reason is that ALA is not found in plasma after topical application and free PpIX and heme are considered to rapidly disappear from healthy tissues in local areas [56]. Therefore, topical applications would further reduce skin phototoxicity and other side effects. However, we should not administer ALA to patients with porphyria, which is caused by a functional disorder or reduced activity of some enzymes involved in heme metabolism [53].

### 4.5. Clinical Application of ALA-PDD

#### 4.5.1. ALA-PDD System

In the field of oral cancers [36,41,44], a modified xenon lamp (D-light-system, Karl Storz, Germany) has been used as a light source. It has a bandpass filter to emit blue-violet light at a wavelength of 375–440 nm. Moreover, an endoscope is equipped with 470-nm-long-pass filters, which transmits 8% of the diffusely back-scattered excitation light with a peak at 450 nm and >98% of fluorescence at wavelengths of 470–800 nm, such as back-scattered blue excitation light, autofluorescence, and red fluorescence. Therefore, this system can detect PpIX fluorescence excited from tumors and autofluorescence from healthy tissues [38,41,44]. In addition, a charge-coupled device (CCD) camera has been used in taking PpIX fluorescence images [41,44].

#### 4.5.2. ALA Application for PDD

Using the ALA-PDD method, 0.4–1% ALA solution is topically administered in the oral cavity via continuous oral rinsing for 15 min, and then PDD using blue-violet light at a wavelength of 375–440 nm is performed after incubation for 1–2.5 h (Table 2). This incubation time is required for the selective accumulation of PpIX in malignant lesions, and it reflects the time at which the difference in the PpIX concentration between malignant lesions and healthy tissues reaches its maximum [38].

#### 4.5.3. Clinical Application of ALA-PDD for Visualizing OED in OPMDs and OSCC

Leunig et al. reported the clinical application of ALA-PDD for detecting OSCC for the first time [36]. They used ALA-PDD in 16 patients with histopathologically confirmed OSCC, and all OSCCs showed red fluorescence. Moreover, they performed ALA-PDD on 58 oral mucosal lesions, including OSCC and suspicious malignant lesions, and demonstrated that ALA-PDD could detect severe and moderate OED and early OSCC as red fluorescence, whereas normal mucosa, hyperplasia, and ulcers exhibited autofluorescence. This strategy resulted in a sensitivity of 99% and specificity of 60% for OSCC, CIS, and severe and moderate OED [38]. These results suggested that ALA-PDD might be useful as an exclusive diagnostic strategy. However, the drawback of ALA-PDD is its low specificity, suggesting a high false-positive rate. One of the factors contributing to the low specificity might be the subjective judgment of whether the lesion excites red fluorescence, especially because it is difficult for examiners to judge weak red fluorescence. In fact, it depends on the observation skills of the examiners. Thus, Zheng et al. tried to quantify the fluorescence images obtained from ALA-PDD in order to improve the diagnostic accuracy [41,42,43]. They measured back-scattered excitation (blue), autofluorescence (green), and PpIX fluorescence (red) in each lesion and healthy tissue simultaneously and calculated the red to green and red to blue intensities. These results revealed that the reduction in autofluorescence and the increase in PpIX fluorescence intensity in malignant lesions correspond to differences in the red to green intensity ratio between malignant and normal tissues. Considering this point, they classified oral mucosa lesions into malignant tissues (severe dysplasia, CIS, and invasive OSCC) and benign tissues (healthy, inflammation, and hyperplasia) and measured the red, green, and blue intensities of oral mucosa lesions on the multicolor fluorescence images. As a result, the red fluorescence (635 nm) intensity of malignant tissues was much stronger than that of benign lesions, whereas the blue fluorescence intensity of malignant tissues was lower than that of healthy tissues. Moreover, the red to blue ratio of malignant tissues was significantly higher than that of benign tissues. Therefore, ALA-PDD combined with the measurement of fluorescence intensity could increase the specificity by 36% (60% to 96%) for separating benign lesions from malignant lesions. Thus, they suggested that the combination could differentiate between benign tissues and oral malignancies.

Sharwani et al. focused on diagnosing oral premalignancy lesions and benign lesions, including inflammatory and hyperplastic tissue changes, using a combination of ALA-PDD and the spectroscopic assessment of fluorescence intensity [44]. Specifically, they separated red and green wavelengths using the RGB system and then calculated the red to green ratio. As a result, the red to green ratios of dysplastic lesions (mild-severe OED and CIS) were significantly higher than those of normal tissues. In fact, the sensitivity and specificity for the discrimination between OEDs and normal tissues were 83–90% and 79–89%, respectively. Furthermore, their data also revealed that the red to green ratio of severe dysplasia and CIS was relatively higher than that of mild OED (*p* < 0.053 and *p* < 0.073, respectively). Therefore, the combination of ALA-PDD and fluorescence spectroscopy can discriminate between OEDs and normal tissues or benign lesions; moreover, this strategy can potentially be used to divide OEDs into high- and low-risk dysplasia. However, large-scale case studies (clinical trials) will be required in the near future to verify that ALA-PDD can discriminate between high- and low-risk dysplasia.

## 5. Issues and Future Perspectives

ALA-PDD along with the qualification of fluorescence can discriminate OSCC and high-risk OPMDs from low-risk OPMDs or benign lesions with high sensitivity. In terms of dysplasia, Speight et al. indicated that the ultimate goal of grading OEDs is to provide patients with the most effective management and care [10]. However, grading OEDs is subjective, and a low level of interobserver consistency is often observed, even when following the WHO classification system [60,61]. In addition, it is generally difficult to distinguish mild dysplasia from reactive and regenerative atypical lesions related to inflammation and ulcerations [14]. Recently, the new WHO classification proposed a binary system that divides OEDs into high-risk and low-risk [5,62]. Liu et al. and Nankivell et al. confirmed that the binary OED grading system had the credibility and reliability for prognostic diagnoses [63,64] and was able to improve the consistency between the grading of OED by each pathologist. Most interestingly and importantly, from previous reports concerning the clinical appliance of ALA-PDD, it was likely to classify OEDs into the presence and absence of red fluorescence, and the lesions with red fluorescence are histologically diagnosed as high-risk dysplasia, whereas most lesions without red fluorescence are categorized as low-risk dysplasia. In other words, discrimination by ALA-PDD might resemble one aspect between high-risk and low-risk dysplasia following the new binary system. By contrast, other light-based systems detect all OEDs as positive, suggesting that these systems do not have the potential to facilitate the grading of dysplasia [18,19]. Therefore, ALA-PDD might be useful for grading OED and providing clinicians with some information in deciding whether a biopsy should be performed on suspicious superficial lesions. However, the binary system diagnoses the lesions based on alternations in both architectural and cytologic features, whereas ALA-PDD depends only on differences in cell metabolic activity. In the future, it is necessary to investigate how ALA-PDD expresses architectural and cytological features of OED.

The histopathological diagnosis of a biopsy depends on the selection of the optimal biopsy area in the lesions. For example, if a biopsy specimen is not obtained from an optimal area of the lesion, especially in a heterogeneous, multifocal, or wide lesion, which likely contains various grades of OED and CIS, the lesions may subsequently undergo malignant transformation into advanced cancer. However, ALA-PDD could help prevent underdiagnosis and/or misdiagnosis associated with a sampling error by identifying the worst area of a lesion as red fluorescence.

The drawback of ALA-PDD is that it produces a few false positives, which in turn results in lower specificity. Hyperplastic or chronic inflammation lesions and radiated mucosa may be observed as red fluorescence [40,43]. This phenomenon has been reported in urology and gastroenterology. Certain chronic inflammations of the bladder urothelial epithelium (urinary tract) [65] and colon [66] have also showed red fluorescence. Inflamed tissues often exhibit a high non-specific accumulation of PpIX. This mechanism might be related to the amount of ferrum (iron) within cells, but the details remain unclear.

On the other hand, false-negative lesions can be high-risk dysplasia or cancerous lesions covered with hyperkeratosis or necrotic tissue [67]. This is because keratinization or necrotic tissues might block the infiltration of ALA and mask the red fluorescence. Currently, there is no effective way to address these lesions with hyperkeratinization for ALA-PDD. To overcome these problems, optical diagnostic techniques such as optical coherence tomography (OCT), probe-based confocal laser endomicroscopy (pCLE), and Raman spectroscopy (RS) might be useful. OCT can delineate the internal structure of tissue by analyzing back-scattered light obtained from a tissue using an optical interferometer [45]. Moreover, pCLE can observe the tissue structure and vasculature of oral mucosa lesions in real time [46]. Therefore, ALA-PDD in combination with OCT and pCLE might clarify alternatives of cell metabolism and the tissue structure of oral mucosa lesions, thereby improving the diagnostic accuracy. Contrarily, RS can highlight the characterization of molecules and their alternatives in cells and tissues by analyzing Raman spectrum, consisting of scattered light of various wavelengths from molecules following irradiation [47]. Thus, RS might identify a slightly different PpIX concentration between normal and malignant cells. We hope that future studies will verify the utilities of these optical diagnostic devices for observation of oral mucosa lesions in clinical settings.

Currently, the ALA-PDD system that is used is an endoscope type. However, all clinics and hospitals do not own this type of device due to relatively poor cost effectiveness. Therefore, a more compact PDD system such as ViziLite^®^ and an auto-fluorescence-based system would be highly convenient. Contrarily, spectroscopic analysis is useful for the objective judgment of ALA-PDD results; however, this analysis is particularly complicated because the optical splitter, spectrometer, and repeated calibration are required to measure fluorescence spectra [67]. Moreover, measuring spectra using a small probe is time-consuming, and the small probe cannot measure the entire lesion, especially those that are widespread because of photobleaching reactions, which is the attenuation of red fluorescence caused by long-term irradiation. Furthermore, it is difficult to measure under the same conditions because the intensity of fluorescence drastically changes depending on the distance between the endoscope tip and lesion surface. Therefore, spectroscopy has been considered to have certain limitations during oral examinations. To overcome these limitations, a novel handheld ALA-PDD system and a simple analysis system should be developed. Altogether, although various technologies and devices have been developed to date, each technology has its strengths and weaknesses. To overcome these problems and establish an ideal technology/device for the accurate diagnosis of OSCC at an early stage and high-risk OPMDs, energetic and interdisciplinary research and clinical trials should be conducted in the future. Moreover, even if useful technology is established or an efficient diagnostic device is developed, it is important that the final differential diagnosis is made carefully by experienced specialists/examiners.

## 6. Conclusions

The improvement of diagnostic techniques for oral mucosa lesions could decrease patient suffering associated with oral cancer. Each light-based diagnostic device can assist in the diagnosis of oral mucosa lesions. Examination under chemiluminescent light could delineate oral lesions because of the improved brightness and sharpness of margins in the identified lesions. Autofluorescence-based systems can discriminate oral cancer and dysplasia from benign lesions in combination with the quantitative analysis of autofluorescence. ALA-PDD may be suitable for detecting small changes or malignant transformation in cells within superficial mucosa lesions that cannot be detected by COEs. In fact, it enables the earliest detection of malignant lesions. Moreover, the combination of the ALA-PDD system and simple imaging processing has been proven to have higher sensitivity and specificity for identifying high-risk dysplasia and cancer and objectively discriminating between high-risk and low-risk OPMDs. Hence, this system might contribute to the application of minimally invasive therapies and improvements in patient prognosis and the longitudinal surveillance of patients with OPMDs.

## Figures and Tables

**Table 1:** The transformation rates of various grades of dysplasia.

Author	Malignant Transformation Rate (%)				Follow up Period (Years)
	Initial Histopathological Diagnosis				
	Severe	Moderate	Mild	No Dysplasia	
Mehanna HM (7)	24.1	10.3 (Moderate + Mild)			0.5–16
Warnakulasuriya S (9)	26.7	15.7	4.8	-	10
	5.6 (annual)	1.8 (annual)		0.3 (annual)	
Speradio M (8)	39.0	18.0	6.0	0.012	5–15

Annual: annual transformation rate.

**Table 2:** Clinical application of ALA-PDD for oral malignant or premalignant lesions.

Author	Subject	Case	Oral Rinse Time (m)	Incubation Time (h)	PDD System	Sensitivity (%)	Specificity (%)
Leunig 1996	Oral squamous cell carcinoma	16	15	1–2	Endoscope type	-	-
Leunig 1996	Neoplastic lesion	11	15	1–2	Endoscope type	-	-
Leunig 2000	Suspicious oral cancer	58	15	1–2.5	Endoscope type	99	60
						for detection of OSCC, CIS, severe and moderate dysplasia	
Betz 2002	Suspicious or malignant Oral and oropharynx lesion	58	15	1–2	Endoscope type Spectroscopy	100	51.3
Zheng 2002	Premalignant or malignant lesion	16	15	1–2	Endoscope type	-	-
					Fluorescence qualification	Discrimination between malignant lesions and benign tissues	
Zheng 2002	Premalignant or malignant lesion	70	15	1–2	Endoscope type	95	97
					Fluorescence qualification	Discrimination between malignant lesions and benign tissues	
Zheng 2004	Suspicious Premalignant or malignant lesion	118	15	1.5–2	Endoscope type Fluorescence qualification	92	96
						for dysplasia	
						98	96
						for cancer	
						Good differentiation between the different stages of dysplasia and cancer	
Sharwani 2006	Suspicious oral leukoplakia	71	15	1–2	Endoscope type Fluorescence qualification	83–90.3	79–89.5
						for dysplasia or CIS	
						Discrimination between normal and dysplastic lesions	

Endoscope type: xenon short-arc lamp for the excitation system, an endoscope equipped with a filter to block the back-scattered excitation fluorescence, and a camera to obtain the fluorescence images. Fluorescence qualification: calculation of the red to green intensity ratio and red to blue intensity ratio. Oral rinse: continuous oral rinsing using ALA solution. Incubation time: the time from the topical administration to PDD.
